# Building a Special Pathogen Response Center from the Ground Up

**DOI:** 10.1017/ash.2024.238

**Published:** 2024-09-16

**Authors:** Brooke Brewer, Natalie Schnell, Emily Sickbert-Bennett Vavalle, David J Weber, David Wohl, William Fischer

**Affiliations:** UNC Health; UNC Hospitals; University of North Carolina at Chapel Hill

## Abstract

**Background:** In September 2022, UNC Hospitals was awarded a Regional Emerging Special Pathogens Treatment Center (RESPTC) grant by the U.S. Department of Health and Human Services Administration for Strategic Preparedness and Response (ASPR) to care for up to two patients with viral hemorrhagic fever, or similar pathogen, and up to ten patients with novel respiratory pathogens. Intensive infection prevention efforts and timely multidisciplinary commitment was required to develop the Space, Strategy, Staff, and Stuff needed to care for patients with a special pathogen. **Methods:** Multiple space needs assessments were undertaken to acquire spaces for the care of patients, simulation training, and a dedicated laboratory. Strategies for developing the response plan required collaboration with hospital executives, nursing leadership, public health leaders, and regional partners. Staff were recruited across various disciplines to join the response team and were provided hands-on skills training which was assessed by post-training surveys. Specialized ‘stuff’ (i.e., PPE, training equipment, and waste management devices) were researched and procured for use by the team. **Results:** Patient care and dedicated laboratory space was identified within existing infrastructure, and renovation plans were developed to adapt the space for these specialized activities. A waste management plan that benefits the hospital for routine waste and allows for Category A waste management was approved. Fifty-three staff members were recruited from 3 main disciplines (RNs, MDs, Paramedics), and across numerous settings (Medicine Acute Care & ICU, Pediatric ICU & Stepdown, Air Care/Transport, Burn ICU, Surgery Stepdown, Emergency Medicine, Infection Prevention, Infectious Disease) were trained during five 4-hour training sessions, culminating in an exercise involving transporting a rule–out Ebola patient to the hospital’s special pathogens unit. Post-training evaluations demonstrated a very high level of confidence (‘strongly agree’) in staffs’ knowledge about the RESPTC site (92.3%), special pathogens (80.8%), collaboration needed for managing patient care (80.8%), and in their comfort with special PPE donning and doffing (73.1%). **Conclusions:** Using a systematic approach to develop Space, Strategy, Staff, and Stuff, a large academic hospital readied itself to become a new RESPTC site. Key lessons learned include the importance of a multidisciplinary response team; local, state, and regional coordination for care planning and delivery; and early community partnership development. Logistical infrastructure and waste management challenges continue to require partnership with hospital leadership to optimize workflows and patient care. Holistic decision-making around infrastructure has led to changes that benefit all hospital patients and offer efficiencies to

**Disclosure:** William Fischer: Consultant - Roche, Merck, Inhalon Biopharma; Speaker for ACGME - IMG. David J Weber: Consultant on vaccines: Pfizer; DSMB chair: GSK; Consultant on disinfection: BD, GAMA, PDI, Germitec

